# 298. Examining the Relationship Between Excess Weight and In-Hospital Mortality in COVID-19 Patients in Southwest Georgia, U.S

**DOI:** 10.1093/ofid/ofab466.500

**Published:** 2021-12-04

**Authors:** Austin C Dykes, Daniel B Chastain, Geren Thomas, Henry N Young, Andrés F Henao Martínez, Carlos Franco-Paredes, Sharmon P Osae

**Affiliations:** 1 University of Georgia, Albany, Georgia; 2 University of Georgia College of Pharmacy, Albany, GA; 3 John D. Archbold Memorial Hospital, Thomasville, Georgia; 4 University of Colorado Anschutz Medical Campus, Aurora, Colorado; 5 University of Colorado Denver, School of Medicine, Aurora, CO

## Abstract

**Background:**

There are multiple mechanisms for the interconnection between obesity and adverse outcomes in COVID-19. Body mass index (BMI) has historically been used to delineate body fatness, but does not include age, which could influence the relationship between body fat and BMI. Ideal body weight (IBW) equations predict a single IBW, which could allow improved recognition of adults with excess weight at increased risk of death from COVID-19. The purpose of our study was to determine whether an association exists between excess weight and in-hospital mortality in COVID-19 patients.

**Methods:**

This was a multicenter, retrospective chart review of hospitalized patients with COVID-19. Patients were separated in two groups based on the difference between actual body weight (ABW) and IBW (ABW/IBW ≤ 120% and ABW/IBW > 120%) to compare rates of in-hospital mortality and length of stay (LOS). A subgroup analysis of patients with ABW/IBW > 120% was conducted to compare in-hospital mortality between patients with ABW/IBW 121-149%, ABW/IBW 150-199%, and ABW/IBW ≥ 200%.

**Results:**

A total of 445 patients were included of which 71% were in the ABW/IBW > 120% group. Patients in the ABW/IBW ≤ 120% group had higher median age (71 [IQR 64-80.5] vs 60 [IQR 50-70] years) compared to those in the ABW/IBW > 120% group. Fewer African Americans and females were in the ABW/IBW ≤ 120% than in the ABW/IBW > 120% group (65% vs 86% and 35% vs 64%, respectively). There was no difference in the rate of in-hospital mortality between patients in the ABW/IBW ≤ 120% and ABW/IBW > 120% group (26% vs 20%, p=0.174). Average LOS was 10.5 days (SD 9.2) for patients in the ABW/IBW ≤ 120% and 9.3 days (SD 9.5) for those in the ABW/IBW > 120% group (p=0.227). Among those in the ABW/IBW > 120% group, in-hospital mortality was 14%, 23%, and 22% in patients with ABW/IBW 121-149%, ABW/IBW 150-199%, and ABW/IBW ≥ 200%, respectively (p=0.192).

**Conclusion:**

In-hospital mortality and LOS were not significantly higher among COVID-19 patients with excess weight, defined by ABW/IBW > 120%, when compared to those with ABW/IBW ≤ 120%. Further research is needed to compare COVID-19 outcomes when BMI and ABW/IBW are used to define excess weight.

**Disclosures:**

**All Authors**: No reported disclosures

